# Aberrant patterns of default‐mode network functional connectivity associated with metabolic syndrome: A resting‐state study

**DOI:** 10.1002/brb3.1333

**Published:** 2019-09-30

**Authors:** Barnaly Rashid, Sheena I. Dev, Michael Esterman, Nicolette F. Schwarz, Tori Ferland, Francesca C. Fortenbaugh, William P. Milberg, Regina E. McGlinchey, David H. Salat, Elizabeth C. Leritz

**Affiliations:** ^1^ Neuroimaging Research for Veterans Center (NeRVe), Geriatric Research Education and Clinical Center (GRECC) VA Boston Healthcare System Boston Massachusetts; ^2^ Harvard Medical School Boston Massachusetts; ^3^ SDSU/UCSD Joint Doctoral Program in Clinical Psychology San Diego California; ^4^ Department of Psychiatry Boston University School of Medicine Boston Massachusetts; ^5^ McLean Imaging Center McLean Hospital Belmont Massachusetts; ^6^ The Athinoula A. Martinos Center for Biomedical Imaging Boston Massachusetts

**Keywords:** default mode network, functional connectivity, metabolic syndrome, resting‐state fMRI, seed‐based analysis

## Abstract

**Introduction:**

Metabolic syndrome (MetS) is a clustering of three or more cardiovascular risk factors (RF), including hypertension, obesity, high cholesterol, or hyperglycemia. MetS and its component RFs are more prevalent in older age, and can be accompanied by alterations in brain structure. Studies have shown altered functional connectivity (FC) in samples with individual RFs as well as in clinical populations that are at higher risk to develop MetS. These studies have indicated that the default mode network (DMN) may be particularly vulnerable, yet little is known about the overall impact of MetS on FC in this network.

**Methods:**

In this study, we evaluated the integrity of FC to the DMN in participants with MetS relative to non‐MetS individuals. Using a seed‐based connectivity analysis approach, resting‐state functional MRI (fMRI) data were analyzed, and the FC measures among the DMN seed (isthmus of the cingulate) and rest of the brain voxels were estimated.

**Results:**

Participants with MetS demonstrated reduced positive connectivity between the DMN seed and left superior frontal regions, and reduced negative connectivity between the DMN seed and left superior parietal, left postcentral, right precentral, right superior temporal and right superior parietal regions, after accounting for age‐ and sex‐effects.

**Conclusions:**

Our results suggest that MetS is associated with alterations in FC between the DMN and other regions of the brain. Furthermore, these results indicate that the overall burden of vascular RFs associated with MetS may, in part, contribute to the pathophysiology underlying aberrant FC in the DMN.

## Graphical abstract

Metabolic Syndrome, the clustering together of cerebrovascular risk factors, is associated with alterations to brain function and structure. In this study, individuals with MetS demonstrated reduced positive connectivity between the default mode network, and critical brain regions.

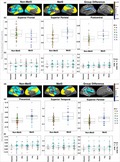

## 1 INTRODUCTION

Metabolic syndrome (MetS) is defined as a clustering of three or more cardiovascular risk factors (RF) that includes hypertension, abdominal obesity, high levels of fasting glucose (i.e., hyperglycemia), and low levels of high density lipoprotein cholesterol (HDL‐C) and high levels of triglycerides (i.e., dyslipidemia) (Grundy, 2005). With overall prevalence continuing to increase every year (Beltrán‐Sánchez, Harhay, Harhay, & McElligott, 2013), MetS is now considered as a substantial threat for the development of vascular‐related cognitive impairment (Van den Berg, Biessels, Craen, Gussekloo, & Westendorp, 2007; Kim & Feldman, 2015; Yaffe, Weston, Blackwell, & Krueger, 2009) and neurodegenerative conditions such as vascular dementia (Panza et al., 2011; Solfrizzi et al., 2011) and Alzheimer's disease (AD) (Misiak, Leszek, & Kiejna, 2012; Raffaitin et al., 2009). Prevalence rates have been estimated to approach 35% of the general U.S. population and increase to 54.7% of older adults over the age of 60, suggesting that older adults are disproportionally affected by the syndrome (Shin, Kongpakpaisarn, & Bohra, 2018). Moreover, given the fact that MetS is highly prevalent in middle age (Aguilar, Bhuket, Torres, Liu, & Wong, 2015; Arai et al., 2010; Grundy, 2008), there is a great need for early detection and intervention in order to prevent or delay cognitive and functional decline. Thus, it is critical to understand the relationship between the factors of MetS and the risk of brain degeneration.

The shared underlying pathophysiological mechanisms of the co‐occurring RFs in MetS interrupt the cerebrovascular mechanism, which may result in disrupted structural and functional integrity in the brain. While researchers have utilized advanced neuroimaging techniques to measure effects of MetS on brain structure (Schwarz et al., 2018) and function (Haight et al., 2015; Kenna et al., 2013), the specific functional domains across the brain and their regional functional connectivity (FC) (Greicius, Krasnow, Reiss, & Menon, 2003; Van Den Heuvel & Pol, 2010) targeted by MetS are still poorly explored. Moreover, while investigators have examined individual RF's contribution to disrupted brain functions and their connectivity within isolation (Chen et al., 2014; Cui et al., 2015; Garcia‐Garcia et al., 2013; Hoogenboom et al., 2014; Musen et al., 2012; Son et al., 2015; Xia et al., 2015), the shared contribution of RFs in MetS needs to be further studied in order to comprehensively understand the “*comorbidity*” impact.

Resting state fMRI provides important measures of brain function and can increase our knowledge of how MetS affects the brain. Exploring the default mode network (DMN) is important due to prior evidence suggesting its role as a biomarker of cognitive function and age‐related decline (Greicius, Srivastava, Reiss, & Menon, 2004; Sorg et al., 2007; Zhou et al., 2010). Indeed, Zhou and colleagues have presented substantial evidence that the DMN is a robust correlate of pathological brain aging (Zhou et al., 2010). The DMN is comprised of anatomically distinct brain regions, including the posterior cingulate cortex (PCC), medial prefrontal cortex (mPFC), and lateral parietal cortices (Fox et al., 2005), that co‐activate during mental rest, and deactivate during tasks with moderate or higher cognitive demand (e.g., episodic memory; Buckner, Andrews‐Hanna, & Schacter, 2008, Daselaar, Prince, & Cabeza, 2004, Fox et al., 2005, Miller et al., 2008, Raichle et al., 2001).

Several studies have linked vascular diseases to disruption in DMN (Damoiseaux & Greicius, 2009; Mayda, Westphal, Carter, & DeCarli, 2011; Papma et al., 2013). While greater DMN activity is commonly associated with being off‐task or resting, recent studies have shown associations between DMN activity and cognition (Spreng, 2012; Vatansever, Menon, Manktelow, Sahakian, & Stamatakis, 2015), including the ability to maintain sustained attention over time (Esterman, Noonan, Rosenberg, & DeGutis, 2012; Fortenbaugh, DeGutis, & Esterman, 2017; Kucyi, Esterman, Riley, & Valera, 2016). Studies have also observed reduced cerebrovascular function (i.e., Cerebral Blood Flow) in DMN regions in AD (Alsop, Detre, & Grossman, 2000; Jagust & D'Esposito, 2009; Johnson et al., 2005), mild cognitive impairment (MCI) (Duschek & Schandry, 2007; Johnson et al., 2005), and in older adults without cognitive impairment (Claus et al., 1998; Jagust & D'Esposito, 2009; Wu et al., 2008). Given the associations between DMN connectivity and cerebrovascular integrity of DMN regions, particularly in aging, it is critical to investigate the effects of MetS on functional connectivity of the DMN. Studies have suggested that the possession of just one individual RF increases risk of developing vascular‐related cognitive impairment and neurodegenerative conditions, such vascular‐related cognitive impairment (Antoniak et al., 2003; Kivipelto et al., 2005; Viswanathan, Rocca, & Tzourio, 2009) and Alzheimer's disease (AD) (Kivipelto et al., 2001, 2005; Luchsinger et al., 2005). Of note, these diseases have also been associated with alterations in DMN functional connectivity (Dennis & Thompson, 2014; Kim et al., 2016). Importantly, risk factors rarely occur in isolation, further highlighting the importance of examining brain integrity in conditions of comorbid risk, such as MetS. We propose that there may be a shared underlying pathophysiological mechanism between the development of neurodegenerative disorders and the component RFs of MetS, and that the presence of MetS increases vulnerability to neural compromise, over and above what has been associated with individual vascular RFs alone.

Recently, functional connectivity (FC) analysis of the resting state blood oxygenation level dependent, or BOLD, fMRI signal has become a powerful neuroimaging tool for identifying disease‐related biomarker (Biswal, Zerrin Yetkin, Haughton, & Hyde, 1995). FC refers to the temporal covariance/correlation of BOLD time series among two or more spatially distinct brain regions. Resting state FC analysis offers the ability to study functional networks without confounding effects of cognitive ability to perform a particular task, making its application appealing to clinical population with cognitive impairment (Auer, 2008; Fox & Raichle, 2007; Greicius, 2008; Rogers, Morgan, Newton, & Gore, 2007). Indeed, disruption in resting state FC patterns have been identified in a wide range of psychiatric and neurodegenerative disorders, including Alzheimer's disease (Greicius et al., 2004; Sorg, Riedl, Perneczky, Kurz, & Wohlschlager, 2009), Parkinson's disease (Helmich et al., 2009; Wu et al., 2009), mild cognitive impairment (Bai et al., 2009; Pihlajamaki, Jauhiainen, & Soininen, 2009; Sorg et al., 2007), schizophrenia (Jafri, Pearlson, Stevens, & Calhoun, 2008; Rashid, Damaraju, Pearlson, & Calhoun, 2014; Repovs, Csernansky, & Barch, 2011), bipolar disorder (Nguyen et al., 2017), and depression (Greicius, 2008). Thus, FC of the DMN may also prove to be a useful marker to elucidate the impact of MetS, as well as comorbid vascular RFs on brain functioning and connectivity.

While there have been a number of studies examining FC in association with individual risk factors, to the best of our knowledge, no studies have exclusively investigated differences in the DMN functional connectivity in those with and without MetS. In this work, we explored the combined effect of the RFs that is causing the altered FC more so than just an individual RF in isolation. The present cross‐sectional study therefore aimed to examine the FC between DMN and other brain networks, in participants with MetS. We hypothesized that compared to individuals with less than three RFs (i.e., non‐MetS), individuals with MetS will demonstrate disrupted resting state connectivity between DMN and whole‐brain functional networks. Results from our analyses will provide valuable information on the mechanisms by which comorbid vascular risk, a strikingly common problem facing middle and older aged adults, influence the integrity of default‐mode network and its functional connectivity.

## 2 MATERIALS AND METHODS

### 2.1 Participants

Seventy‐eight participants agreeing to undergo structural and functional MRI participated in this study. Twenty‐seven participants were identified with metabolic syndrome (i.e., having three or more RFs; see section 2.41) (**MetS**, age (mean ± *SD*): 65.70 ± 7.87, 3 females), 25 participants without any RFs were identified as healthy controls (HC, age (mean ± *SD*): 59.24 ± 8.83, 11 females), and 26 participants with one or two RFs were identified as premetabolic syndrome individuals ( i.e., 0<RF<3) (**pre‐MetS**, age (mean ± *SD*): 60. ± 8.52, 8 females). Furthermore, jointly the HC and pre‐MetS individuals are referred to as the non‐MetS group in the context of the group analyses.

Table 1 displays group‐wise demographics and characteristics of the participants. Individuals were enrolled from direct clinic recruitment in VA Boston clinics to target those at high risk for MetS, as well as through advertisement in the greater Boston, Massachusetts (USA) metropolitan area. Inclusion criteria required participants to be English speakers and between the ages of 30–90. Participants were excluded for the following reasons: a history of head trauma of moderate to severe severity (e.g., loss of consciousness greater than 30 min, diagnosis of any form of dementia, past or current history of severe psychiatric illness, history of major surgery (e.g., brain or cardiac), significant neurologic illness, history or current diagnosis of drug abuse or dependence. Exclusion criteria also included any contraindication to magnetic resonance imaging (MRI), such as a pacemaker or other metal implant.)

**Table 1:** Group‐wise participant characteristics

A	HC (*N* = 25)	MetS (*N* = 27)	Significance tests
Age (years)	59.24 ± 8.83	65.70 ± 7.87	Student's *t*(50) = −2.79, *p* = 0.007*
Sex (female/male)	11/14	3/24	Fisher's exact test: *p* = 0.011*
Waist circumference (cm)	81.96 ± 9.16	105.26 ± 15.92	Student's *t*(50) = −6.40, *p* = 5.215e−08**
Triglycerides (mg/dL)	72.12 ± 25.87	105.70 ± 43.96	Welch's *t*(42.62) = −3.39, *p* = 0.001*
HDL‐C (mg/dL)	67.52 ± 16.41	51.04 ± 14.84	Student's *t*(50) = 3.80, *p* = 0.0003**
Systolic BP (mm Hg)	111.12 ± 9.66	131.52 ± 14.71	Student's *t*(50) = −5.86, *p* = 3.623e−07**
Diastolic BP (mm Hg)	65.80 ± 9.35	76.19 ± 8.34	Student's *t*(50) = −4.23, *p* = 9.803e−05**
Fasting blood glucose (mg/dL)	86.68 ± 10.02	104.59 ± 14.42	Welch's *t*(46.49) = −5.23, *p* = 3.9e−06**

B	Pre‐MetS (*N* = 26)	MetS (*N* = 27)	Significance tests
Age (years)	60 ± 8.52	65.70 ± 7.87	Student's *t*(51) = −2.53, *p* = 0.014*
Sex (female/male)	8/18	3/24	Fisher's exact test: *p* = 0.1
Waist circumference (cm)	97.18 ± 13.69	105.26 ± 15.92	Student's *t*(51) = −1.9776, *p* = 0.053
Triglycerides (mg/dl)	109.58 ± 56.13	105.70 ± 43.96	Welch's *t*(47.365) = 0.279, *p* = 0.7815
HDL‐C (mg/dl)	56.54 ± 16.15	51.04 ± 14.84	Student's *t*(51) = 1.29, *p* = 0.20
Systolic BP (mm Hg)	133.19 ± 16.76	131.52 ± 14.71	Student's *t*(51) = 0.39, *p* = 0.70
Diastolic BP (mm Hg)	79.81 ± 8.04	76.19 ± 8.34	Student's *t*(51) = 1.609, *p* = 0. 0.11
Fasting blood glucose (mg/dl)	96.92 ± 16.73	104.59 ± 14.42	Welch's *t*(49.30) = −1.78, *p* = 0.08

Note

Continuous variables are presented as mean ± standard deviation. cm = centimeter; mg/dL = milligrams per deciliter; HDL‐C = high‐density lipoprotein cholesterol; mm Hg = millimeters of mercury.

* *p* < 0.05;

** *p* < 0.001.

The study was approved and monitored by the Institutional Review Board of the Veterans Administration Boston Healthcare System (VA), Jamaica Plain, MA, USA. All participants provided informed consent prior to study procedures.

### 2.2 Risk factor measurement

Fasting blood (12 hr) was drawn and processed for analysis of serum RF levels including triglycerides, HDL‐C, and fasting plasma glucose. Systolic and diastolic blood pressure (BP) were recorded in a seated position after five minutes of rest with the arm at rest, at the level of the heart, using a standard sphygmomanometer. A second measurement was obtained five minutes later and the average of two values was recorded. Waist circumference measurement was taken while standing, to the nearest centimeter, with measuring tape placed around the abdomen at the level of the umbilicus. Medications taken to treat hypertension, diabetes, or abnormal cholesterol were reported by participants and recorded by an examiner.

### 2.3 Metabolic syndrome assessment

MetS criteria were determined from the RF measures. Participants with MetS were defined as meeting thresholds for three or more of the following component RFs: (a) elevated waist circumference ≥102/88 cm (male/female), (b) elevated triglycerides ≥150 mg/dl or drug treatment for dyslipidemia, (c) reduced HDL‐C <40/50 mg/dl (male/female) or drug treatment for dyslipidemia, (d) elevated systolic BP ≥130 mm Hg or diastolic BP ≥85 mm Hg or drug treatment for hypertension, and (e) elevated fasting plasma glucose ≥100 mg/dl or drug treatment for elevated glucose or diabetes (Grundy, 2005).

### 2.4 Imaging data acquisition

For 70 participants, neuroimaging data were acquired on a 3‐Tesla Siemens, Erlangen, German Prisma Fit 60 cm bore (RF coil ID) using a transmission body coil and a 32‐channel reception head coil.

The first eight participants were scanned prior to the scanner upgrade, on a 3‐Tesla Siemens (Erlangen, Germany) TIM Trio scanner, using a transmission body coil and a 32‐channel reception head coil. Two MPRAGE (Magnetization Prepared Rapid Gradient Echo) T1‐weighted structural scans (repetition time (TR)/echo time (TE): 2,530/3.35 ms, flip angle = 7°, inversion time = 1,200 ms, field of view (FOV) = 256 × 256 mm, acquisition matrix 256 × 256, 176 contiguous sagittal slices, voxel size = 1 × 1 × 1 mm) were acquired for surface reconstruction, FC seed placement, and inter‐participant registration. Resting state functional data were acquired in a single run (gradient echo echo‐planar imaging (EPI), TR/TE: 4,000/31 ms, flip angle: 90°, FOV = 128 × 128 mm, voxel size 2 × 2 × 2.5 mm, 55 axial slices, 90 volumes, 6:12 min per run). To achieve a T1 steady state, the scanner was set to automatically discard the first three volumes from the acquired data. Prior to scanning, participants were instructed to keep their eyes open and stay awake.

### 2.5 Imaging data preprocessing

A model of each subject's cortical surface was reconstructed from the T1 ‐weighted MRI volume using FreeSurfer as described previously (Dale, Fischl, & Sereno, 1999; Lindemer, Salat, Leritz, McGlinchey, & Milberg, 2013). The surface was then anatomically parcellated into 34 distinct ROIs (which included the seed region) using the Desikan‐Killiany atlas (Desikan et al., 2006; Fischl et al., 2004). Functional neuroimaging data were processed using a combination of FreeSurfer (Fischl, Sereno, Tootell, & Dale, 1999), AFNI (Cox, 1996), and FSL (Jenkinson, Beckmann, Behrens, Woolrich, & Fsl, 2012) based on the FSFAST processing stream (http://freesurfer.net/fswiki/ FsFast). Resting state fMRI scans for each subject were preprocessed using a standard stream (motion correction using six parameters, time shifting, concatenation of scans, motion regressed from time series, regression of the global mean, and the average time courses from the white matter and the ventricles, band pass filtering between 0.01 and 0.1 Hz). Time points with excessive motion were excluded (0.5 mm/TR). Data were sampled to and smoothed on the surface, and each brain was warped to a surface‐based template (fsaverage) (Fischl, Sereno, & Dale, 1999). Seed regions were derived from surface‐based parcellation of the cortex (Fischl et al., 2002). Previous studies have shown the isthmus cingulate region to be a reliable seed to study the default network (Poole et al., 2016; Robinson et al., 2015; Seibert & Brewer, 2011). Thus, the bilateral superior third of the isthmus of the cingulate, as defined within each participant's native space, was used as a seed region. Following the FreeSurfer FSFAST processing stream, the vertex‐wise partial correlation to the DMN seed was computed and used for further group‐level analyses. Briefly, to estimate FC to the DMN seed, the mean time series of the DMN seed was first correlated with all other voxels’ time series in the brain, and then the measures were transformed onto the cortical surface and represented them as vertex‐wise partial correlation (i.e., at each vertex over the cortical surface, more details can be found in FS‐FAST processing stream: http://freesurfer.net/fswiki/FsFast).

### 2.6 Statistical analyses

Group differences in age, waist circumference, triglycerides, HDL‐C, systolic and diastolic blood pressure (BP), and fasting blood glucose were examined either with independent two‐sample Student's *t *tests (for normally distributed data as assessed with a Shapiro‐Wilk test), the Wilcoxon rank‐sum test with continuity correction (for non‐normally distributed data), or the Welch's *t *test (when there was a significant group difference in variance as assessed with an *F *test). To examine group differences on categorical variables such as gender, Fisher's exact test was employed. Statistical analyses were two tailed with an alpha level set at *p* < 0.05 and carried out in R (Version 3.2.2).

Group differences and associations (see Table 2) were computed at each vertex over the cortex using multiple linear regression using FreeSurfer's mri_glmfit function, and custom scripts in MATLAB (Mathworks; Natick, MA). The nuisance regressors of age, sex, and scanner were included in all models unless otherwise noted. Family‐wise corrections for multiple comparisons were simulated with pre‐computed Monte‐Carlo simulations of 10,000 iterations using FreeSurfer's mri_glmfit‐sim function, with vertex‐ and cluster‐wise thresholds of *p* **< **0.05.

**Table 2:** Group‐wise risk factors

RF Criteria	Non‐MetS (*N* = 51)	MetS (*N* = 27)
i) Waist circumference ≥102/88 cm (male/female)	13	18
ii) Triglycerides ≥150 mg/dl	3	6
iii) HDL‐C <40/50 mg/dl (male/female)	5	7
iv) Systolic BP ≥150 mm Hg or diastolic BP ≥85 mm Hg	18	16
v) Fasting plasma glucose ≥100 mg/dl	7	18

Abbreviations: cm = centimeter; mg/dL = milligrams per deciliter; HDL‐C = high‐density lipoprotein cholesterol; BP = blood pressure; mm Hg = millimeters of mercury.

## 3 RESULTS

### 3.1 Group differences in participants’ characteristics

#### 3.1.1 Demographics

The MetS group was significantly older (*p* = 0.007) and had a larger proportion of males (*p* = 0.011) relative the HC group (Table 1A). Furthermore, the MetS group was significantly older (*p* = 0.014) than the pre‐MetS group, although no significant difference in sex was observed (*p* = 0.1).

#### 3.1.2 Risk factors

Table 2 presents the summary of RFs by group. In the HC group, 25 participants (*HC^N0^*) did not meet the threshold for any RF, 11 participants (*pre* – *MetS^N1^*) met threshold for only one RF, and 15 participants (*pre* – *MetS^N2^*) had two RFs. Elevated blood pressure (BP) was the most common RF (*N* = 18/35%) observed, followed by waist circumference (*N* = 13/25%), glucose (*N* = 7/14%), HDL‐C (*N* = 5/10%), and triglycerides (*N* = 3/6%).

Subsequently, from the MetS group, 12 participants (*MetS^N3^*) met criteria for three RFs, 10 participants (*MetS^N4^*) met criteria for four RFs, and five participants (*MetS^N5^*) met criteria for five RFs. Waist circumference (*N* = 18/67%) and glucose (*N* = 18/67%) were most often met, followed by BP (*N* = 16/59%), HDL‐C (*N* = 7/26%) and triglycerides (*N* = 6/22%). Consistent with prior literature and in expected directions, the HC and MetS groups significantly differed in all measures of RFs (i.e., waist circumference, triglycerides, systolic and diastolic BP, HDL‐C, and glucose (Table 1)). No significant group differences between pre‐MetS and MetS RFs’ measures were observed.

### 3.2 Group differences in functional connectivity

**Figure 1:**
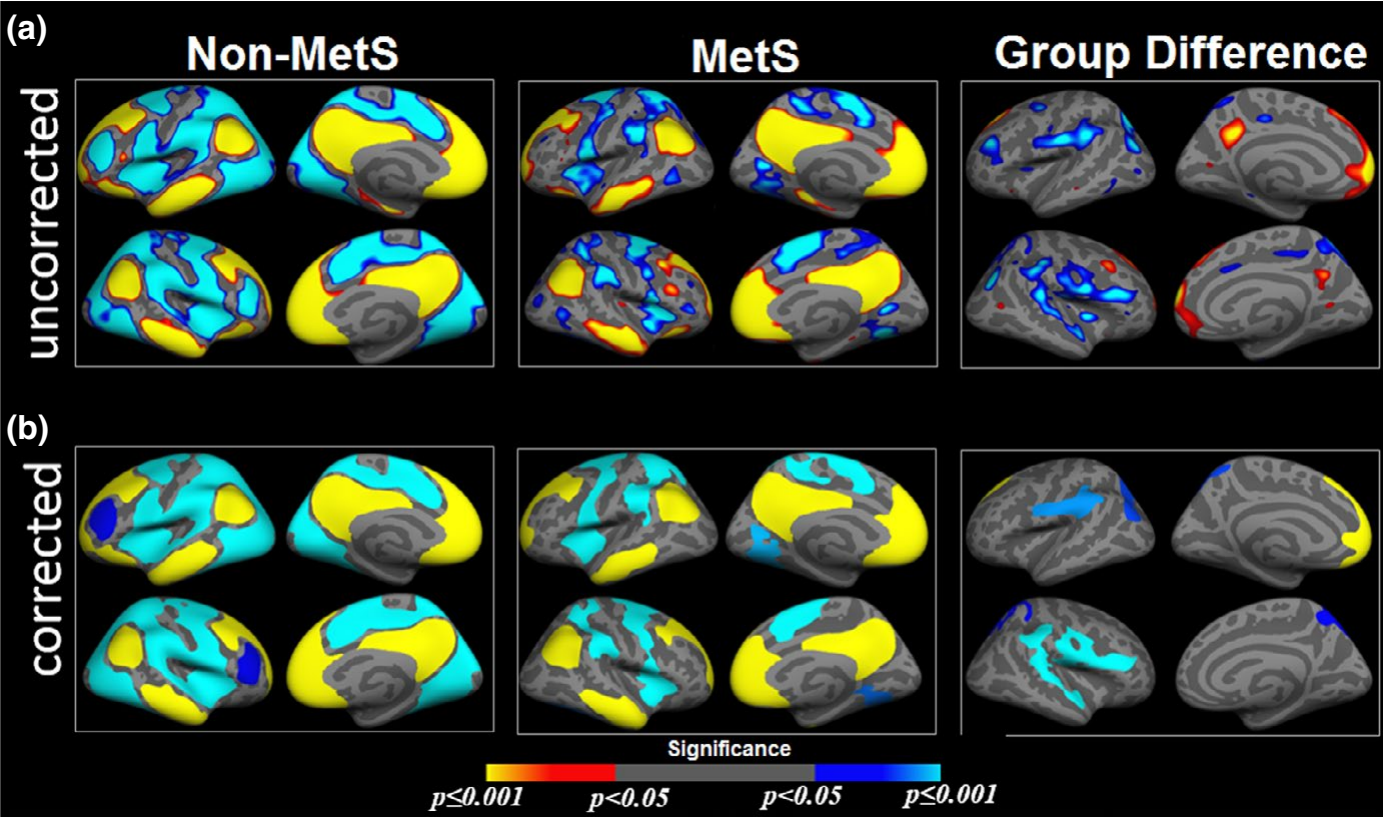
Group difference in functional connectivity. One‐sample group mean (OSGM) results from functional connectivity analyses for non‐metabolic syndrome (Non‐MetS; number of risk factors (RFs) <3) and patients with metabolic syndrome (MetS; RF ≥3), and their group differences without correction (a) and after corrected for multiple comparisons (b). For Non‐MetS group, samples have number of RFs ranging from zero to two, and for metabolic syndrome (MetS) group, samples have three or more RFs

Figure 1 highlights both the uncorrected (Figure 1a) and corrected for multiple comparisons (Figure 1b) results from the GLM analysis showing the one‐sample group mean (OSGM) measures of non‐MetS and MetS groups, and the group differences in FC of the DMN seed to the vertices in the cortex, after regressing out age and sex‐effects. Note that, no significant age‐ and sex‐effects on FC of the DMN seed to the rest of the brain regions was observed after correcting for multiple comparisons (*p* < 0.05). Table 3 provides the mean FC, number of vertices, cluster size (in mm^2^) maximum t‐value, and MNI coordinate for each of the significant clusters from right and left hemispheres. The primary whole‐brain between‐group GLM analysis revealed three clusters in the left hemisphere and three clusters in the right hemisphere that survived multiple comparison correction. Relative to the non‐MetS group, in the left hemisphere the MetS group demonstrated significantly reduced positive correlation between the DMN seed (isthmus cingulate) and left superior frontal region, and significantly reduced negative correlation between the seed and left superior parietal, and left postcentral regions. Also, in the right hemisphere the MetS group showed significantly reduced negative correlation between the seed and right precentral, right superior temporal, and right superior parietal regions. Furthermore, we examined the effects of age and sex on the FC measures and found no significant effects after correcting for multiple comparisons.

**Table 3:** Summary information on clusters that survived multiple comparison correction in general linear model (GLM) analysis

Cortical region	Mean FC (mean ± std)		Number of vertices	Cluster size (mm^2^)	Maximum **t**‐value	MNI (x, y, z)
	Non‐MetS	MetS				
LH						
Superior frontal	0.31 ± 0.14	0.18 ± 0.15	5,353	3,222.12	5.578	−10.3, 57.4, 5.2
Superior parietal	−0.07 ± 0.15	0.09 ± 0.13	4,398	2,329.34	−5.501	−16.9, 71.5, 41.4
Postcentral	−0.16 ± 0.13	−0.03 ± 0.14	6,182	2,656.18	−3.871	−58, 17.7, 27.7
RH						
Precentral	−0.03 ± 0.042	0.01 ± 0.05	8,416	3,897.77	−3.968	58.6, 6, 20
Superior temporal	−0.04 ± 0.05	−0.01 ± 0.06	7,036	3,226.02	−3.481	60.3, −35.1, 16.2
Superior parietal	−0.07 ± 0.07	−0.003 ± 0.10	4,639	2,184.63	−2.662	9.5, −64.6, 59.3

Note

Mean functional connectivity (FC) measures are presented as mean ± standard deviation. mm = millimeter; LH = left hemisphere; and RH = right hemisphere.

### 3.3 Number of RFs and group difference in connectivity

**Figure 2:**
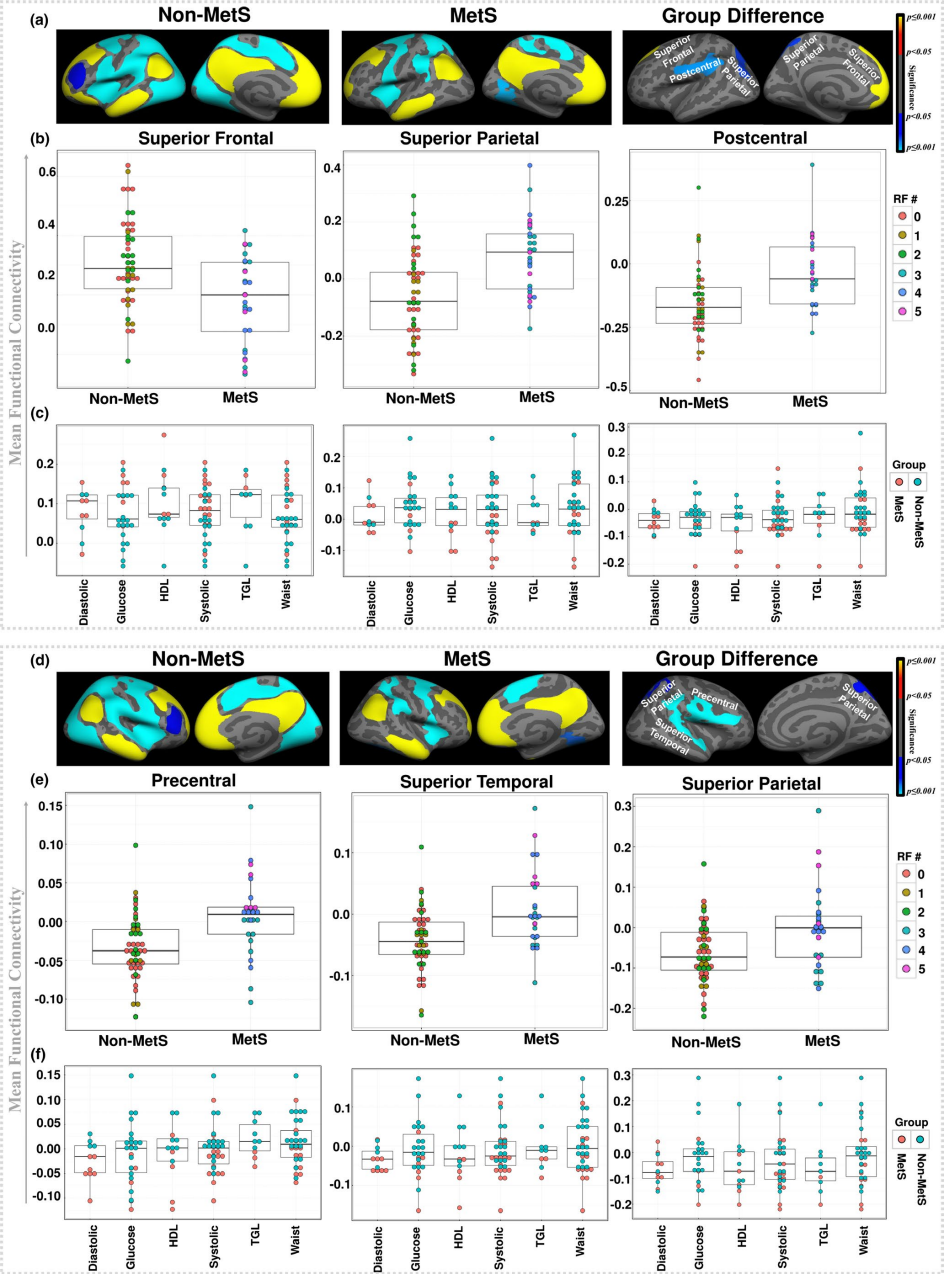
Mean connectivity and risk factors. (a) Results from one‐sample group mean (OSGM) for nonmetabolic syndrome (Non‐MetS) and patients with metabolic syndrome (MetS) and significant left hemisphere's clusters from their group differences after corrected for multiple comparisons, and (b) boxplots showing the group‐wise mean functional connectivity (FC) in each significant cluster and the associated distributions of number of risk factors (RFs) in the left hemisphere. (c) Boxplots showing the mean FC across individual RFs within each significant cluster in the left hemisphere. (d) Results from one‐sample group mean (OSGM) for non‐MetS and patients with MetS and significant right hemisphere's clusters from their group differences after corrected for multiple comparisons, (e) boxplots showing the group‐wise mean FC in each significant cluster and the associated distributions of number of RFs in right hemisphere, and (f) boxplots showing the mean FC across individual RFs within each significant cluster in the right hemisphere. Diastolic: diastolic blood pressure (mm Hg); Glucose: fasting blood glucose (mg/dl); HDL: HDL‐C (mg/dl); Systolic: systolic blood pressure (mm Hg); TGL: triglycerides (mg/dl); and Waist: waist circumference (cm)

Figure 2 presents the group‐wise comparisons for mean regional connectivity in the left and right hemispheres’ clusters that survived multiple comparison correction from GLM analysis (Figure 2a,d), the distribution of the number of RFs across each group (Figure 2b,e), and the cluster‐wise mean FC across individual RFs (Figure 2c,f). In the case of the mean FC between the seed and left superior frontal cluster, where non‐MetS showed significantly greater FC compared to MetS, relatively higher mean FC measures in the non‐MetS group were observed in participants with no RFs or only one RF. The patterns associated with the number of RFs in MetS groups were evenly distributed, suggesting reduced FC in this cluster could result from any number of RFs (in this case the number of RF is three or greater). Similar patterns were observed in FC in the remaining two clusters. Furthermore, Figure 2c and Figure 2f highlight the boxplots showing the mean FC across individual RFs within each significant cluster in the left and right hemispheres, respectively. These results show that the mean FC measures are sparsely distributed across each of the RFs.

### 3.4 Additional validation: no RFs versus three or more RFs

**Figure 3:**
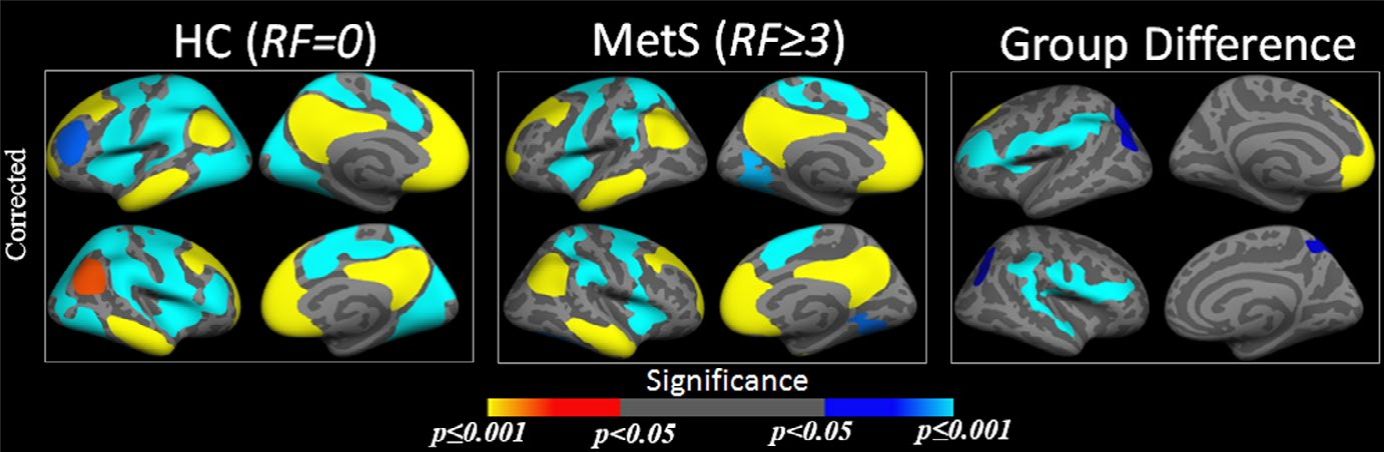
Group difference in functional connectivity (no risk vs. metabolic syndrome). One‐sample group mean (OSGM) results from functional connectivity (FC) analyses for healthy control (HC) without any risk factors (RFs) (*N* = 25) and patients with metabolic syndrome (MetS) with three or more RFs (*N* = 27), and their group differences after corrected for multiple comparisons

In an effort to further validate our findings, and to examine the two groups while balancing the sample size, we conducted an additional GLM analysis in which we compared FC between the MetS group and in a subset of HC participants with zero RFs (HC^N0^ = 25, age (mean ± *SD*): 59.24 ± 8.83, 11 females) (Figure 3).

Even after excluding the participants with one and two RFs from the HC group, between‐group differences in FC were observed in the same clusters as from our primary GLM analysis. Moreover, stronger group difference in FC was captured in the left postcentral cluster, where patients with MetS showed less negative connectivity to the seed (isthmus cingulate) compared to that of the HC group with no RFs.

## 4 DISCUSSION

In this study, we performed a resting state seed based functional connectivity (FC) analysis to evaluate the patterns of connectivity between the default mode network (DMN) and the voxels in the rest of the brain. Previous connectivity studies suggest that typically in HC individuals, the DMN is positively correlated within itself and mostly negatively correlated (i.e., anti‐correlated) with other task‐positive brain networks (Fox et al., 2005). In accordance with these findings, our study found that when compared with the non‐MetS group, the MetS group demonstrated significantly reduced positive correlation between left superior frontal region to the seed (isthmus cingulate), and reduced negative correlation between left superior parietal, left postcentral, right precentral, right superior temporal, and right superior parietal regions to the seed region. These findings demonstrate that MetS may be associated with disrupted resting state FC in primary regions within the DMN. Interestingly, results from our group analysis between the subset of HC participants without any RFs and MetS participants also demonstrated significant group differences in the same regions (with the same directionality in positive and negative connectivity within the non‐MetS and MetS groups) as observed in the main analysis, confirming that FC differences in MetS remain consistent when compared with HC group with zero risk factors. Moreover, as illustrated in Figure 2c, f, the mean FC measures are sparsely distributed across each of the RFs, without showing any trends for significant influence of any particular RFs. This might imply that, the co‐occurring RFs in MetS have unique contributions that underlie the aberrant DMN connectivity patterns in MetS individuals, which are not evident from the isolated contributions of the individual RFs.

Additionally, we have performed the group difference analyses for the whole sample following the same processing pipeline as the main analyses without global signal regression. Results for the group difference without global signal regression showed similar patterns as observed in the main analyses (Figure 1). Furthermore, all of the regions from the main analyses showing group differences (i.e., cluster‐wise corrected group difference as seen in Figure 1 with global signal regression) were also observed from the uncorrected group difference maps estimated without global signal regression, with regions just falling below the threshold. We speculate that, since global signal regression accounts for individual variability, without global signal regression, the data will contain greater variability across individuals. Therefore, for the current dataset, the differences observed between with and without global signal regression are due to lack of statistical power, and not influenced by the global signal.

Our novel findings demonstrate altered resting state connectivity in individuals with MetS. These results suggest that multiple co‐occurring vascular RFs may disrupt fundamental brain networks, particularly in frontal, parietal, and temporal regions. This is critical, as it demonstrates that it is likely the *comorbidity* of RFs in MetS that results in this disruption, over and above what is seen when looking at individual RFs separately. Given the fact that most RFs do not occur in isolation, our results provide important evidence of the underlying functional network disruption that we believe is specific to MetS. Future studies will determine if there are particular combinations of MetS risk factors that are more detrimental to disrupted functional connectivity.

From a mechanistic standpoint, it is possible that the collective burden of vascular RFs that comprise the MetS syndrome impact underlying brain vasculature, thus globally disrupting the resting state signals within and across functional neural networks. This interpretation suggests that the damaging effects of MetS on brain function are, at least in part, explained by abnormalities in the brain's vascular system. Indeed, the DMN appears to be particularly vulnerable to neurovascular compromise, and studies have demonstrated reduced cerebrovascular function in the brain regions overlapping this network (Claus et al., 1998; Dai et al., 2009; Johnson et al., 2005), providing additional support for our speculation. Our findings, together with the previous evidence, indicate that disruption in DMN connectivity, a network of primary interest in aging, neurological disease, and psychiatric disorders, (Dunn et al., 2014; Sperling, 2007), may be in part due to underlying changes to the brain's vascular system.

Reduced functional anti‐correlation or negative connectivity between isthmus cingulate and left superior parietal, left postcentral, right precentral, right superior temporal, and right superior parietal regions in MetS may reflect an inability of these participants to appropriately activate DMN or deactivate task positive networks at rest. Activation of DMN at rest is thought to support self‐reflective processing as well as internally directed cognitive functions such as intrinsic attention, remembering autobiographical information, planning and personal future, and perspective taking. (Andrews‐Hanna, 2012; Buckner et al., 2008). Based on our findings, it is possible that individuals with MetS may have dysfunctional brain networks, and therefore exhibiting less modularity in FC. Furthermore, previous evidence suggests that the significance of the DMN‐task positive anticorrelations have been implicated in cognitive functioning, which tend to become weaker in neurodegenerative disorders (Hafkemeijer, Grond, & Rombouts, 2012). Although, future studies are required to fully disentangle the impact of disrupted network connectivity on cognitive tasks in individuals with MetS.

Our findings are consistent with previous studies demonstrating alterations in DMN in the context of one or more of these component RFs. For example, one study reported increased connectivity among anterior and decreased connectivity among posterior DMN networks in patients with diabetes (Cui et al., 2015). Decreased connectivity in posterior nodes was also associated with worse performance on tasks of memory and executive functioning. Animal studies have also observed alterations in the DMN in rats genetically predisposed to develop hypertension (Huang et al., 2016). Another study examined the association between dynamic functional network connectivity and metabolic risks using a sliding‐window clustering approach, and found that metabolic risk was associated with the relative amount of time allocated to dynamic connectivity states (Viviano, Raz, Yuan, & Damoiseaux, 2017). However, these studies did not investigate MetS specifically, and therefore did not determine the aggregate impact of the entire constellation of RFs associated with this syndrome. The present investigation demonstrates that altered FC in individuals who have incurred enough RFs to qualify for MetS. Interestingly, our analyses indicated that a greater number of RFs did not impact FC in the MetS group. Therefore, this study also suggests that simply meeting criteria for MetS imposes enough burden to exert an impact on FC of the DMN to other brain regions.

Alterations in within‐network DMN connectivity has been linked to older age and worse cognition. For example, Huang and colleagues demonstrated that older age was associated with decreased connectivity among ventral nodes of the DMN (Huang et al., 2015). Others have shown associations between within‐network FC of the DMN and poorer performance on tasks of executive functions (including working memory and cognitive set shifting) (van Eimeren, Monchi, Ballanger, & Strafella, 2009; Sambataro et al., 2010) in elderly samples. Andrews‐Hanna and colleagues have observed reduced connectivity between PCC and mPFC (within DMN network nodes) related to worse performance in memory, executive functioning, and processing speed in a healthy aging cohort (Andrews‐Hanna et al., 2007). Taken together, these studies suggest that the DMN is particularly sensitive to pathophysiological changes that can accompany the aging process. Our findings extend upon this literature by also demonstrating alterations between the DMN and out‐of‐network regions in samples of participants that meet criteria for MetS, a syndrome that is more prevalent in older adults. It is possible that this represents an uncoupled synchronization of distinct neural networks at rest, which may play an essential role in cognitive performance or in switching between mental states (i.e., task‐positive vs. task‐negative). Therefore, further studies examining the relationship between functional connectivity of brain networks and cognition, particularly within the cognitive domains that are vulnerable to both vascular RFs and aging, is warranted.

Our findings are also consistent with prior studies that demonstrate alterations in brain structure and function in the presence of MetS or its component RFs (Friedman et al., 2014). Overwhelmingly, these previous studies have demonstrated lower white matter integrity (Salat et al., 2012; Williams et al., 2013), gray matter volume (Kharabian Masouleh et al., 2018) and cortical thickness (Schwarz et al., 2018; Tchistiakova, Anderson, Greenwood, & MacIntosh, 2014), white matter volume (Figley, Asem, Levenbaum, & Courtney, 2016; Karlsson et al., 2013), task related BOLD response (Hoth et al., 2011), and cerebral blood flow (Birdsill et al., 2013) in samples that meet criteria for MetS or possess elevations in one or more RFs. Our findings of altered functional connectivity between the DMN seed and other brain regions extend upon this literature to suggest that the underlying pathophysiology of MetS may also include disrupted connectivity among functional networks in the brain.

Several experimental and methodological limitations must be considered while interpreting the results of this study. First, relative to the non‐MetS group, the number of subjects in MetS group is considerably lower. Indeed, inclusion of more MetS participants would increase the power of statistical analyses, and ultimately provide more robust measures of group difference. Furthermore, the cross‐sectional design of the study limits the interpretation of our findings from a time‐varying perspective. Also, for each subject, the resting‐state functional data consist of only 90 volumes acquired during the 6:12‐min scanning. Since the resting‐state fMRI connectivity estimates are affected by the scan length (Birn et al., 2013), future FC analyses in MetS cohorts should include longer scanning duration and/or higher number of fMRI volumes using a shorter TR in order to reliably interpret the findings. In order to identify how MetS impacts functional brain connectivity over time, it is necessary to examine samples from a longitudinal study design with information on duration of specific RFs. Moreover, given the well‐documented health disparities across different ethnic groups in the context of metabolic syndrome, future studies should also disentangle differential impact on brain health in other ethnic groups. Finally, in this study we examined a single seed, the DMN, and explored its FC to other voxels in the brain. Further consideration of other networks and ROIs will be important to understanding the full impact of MetS on brain functioning.

## 5 CONCLUSION

In summary, MetS is associated with disrupted DMN functional connectivity, which include both positive and negative connectivity with frontal, parietal, and temporal regions. Moreover, even after only including HC participant with no RFs for group difference analyses, altered FC measures between the DMN seed and other brain regions were observed across similar regions as found in our main analysis. This reinforces the fundamental hypothesis of MetS criteria, where a participant is characterized as MetS only if three or more vascular RFs are reported. Also, our results showed that the mean connectivity across MetS group is not distributed based on the number of RFs for a given participant. In other words, our findings do not indicate that having three (lowest possible number) or five (highest possible number) RFs will result in higher (or lower) mean FC. This may suggest that the disruption in FC between DMN and other brain regions may arise from the underlying pathophysiology of MetS, regardless of the number of RFs. The findings from this study may allow the development of functional connectivity based biomarkers as observed between the DMN and other critical brain regions in MetS participants, which could facilitate diagnosis, targeted intervention and, in some cases, prevention of the disease.

## CONFLICT OF INTEREST

The authors declare no competing financial interests in relation to the work presented.

## Data Availability

The data that support the findings of this study are available on request from the corresponding author. The data are not publicly available due to privacy or ethical restrictions (funding agency: National Institute of Neurological Disorders and Stroke, grant number: NS086882, PI: Elizabeth C. Leritz, study title: Neuroimaging and neuropsychological biomarkers of vascular risk factors).
